# High-Pressure Synthesis and Chemical Bonding of Barium Trisilicide BaSi_3_

**DOI:** 10.3390/ma12010145

**Published:** 2019-01-04

**Authors:** Julia-Maria Hübner, Lev Akselrud, Walter Schnelle, Ulrich Burkhardt, Matej Bobnar, Yurii Prots, Yuri Grin, Ulrich Schwarz

**Affiliations:** Max-Planck-Institut für Chemische Physik fester Stoffe, Nöthnitzer Straße 40, 01187 Dresden, Germany; Julia.Huebner@cpfs.mpg.de (J.-M.H.); Lev.Akselrud@cpfs.mpg.de (L.A.); Walter.Schnelle@cpfs.mpg.de (W.S.); ulrich.burkhardt@cpfs.mpg.de (U.B.); matej.Bobnar@cpfs.mpg.de (M.B.); Yurii.Prots@cpfs.mpg.de (Y.P.); Juri.Grin@cpfs.mpg.de (Y.G.)

**Keywords:** high-pressure high-temperature synthesis, barium, silicon, chemical bonding

## Abstract

BaSi_3_ is obtained at pressures between 12(2) and 15(2) GPa and temperatures from 800(80) and 1050(105) K applied for one to five hours before quenching. The new trisilicide crystallizes in the space group *I*$\bar{4}$*2m* (no. 121) and adopts a unique atomic arrangement which is a distorted variant of the CaGe_3_ type. At ambient pressure and 570(5) K, the compound decomposes in an exothermal reaction into (*hP3*)BaSi_2_ and two amorphous silicon-rich phases. Chemical bonding analysis reveals covalent bonding in the silicon partial structure and polar multicenter interactions between the silicon layers and the barium atoms. The temperature dependence of electrical resistivity and magnetic susceptibility measurements indicate metallic behavior.

## 1. Introduction

The Zintl-Klemm concept [1,2] constitutes a powerful framework for understanding the interdependence of chemical bonding and electron count of a rich variety of binary phases formed by element semiconductors such as silicon or germanium with electropositive partners of the alkaline-, alkaline earth- and rare-earth metal groups. Counting rules for compounds such as Ba_2_Si [3], Ba_3_Si_4_ [4] and BaSi_2_ [5] work successfully when a complete charge transfer from the electropositive metal to the tetrel atoms is assumed. The formation of covalent two-center two-electron interactions in the resulting polyanionic partial structures yields an electron-precise electron balance. Phases violating the classical electron count because of unusual coordination environments in the covalent partial structure of the *p*-block element exhibit more exotic bonding properties, often in combination with metal-type electrical conductivity [6,7]. Quite a few of these so-called covalent metals are accessible by high-pressure high-temperature synthesis.

A systematic study of tetrel connectivities in polyanions revealed a variety of motifs with composition *MT*_3_ (*M* = Ca, Sr, Ba, Y, La, Ce, Eu, Gd, Tb, Ho, Er, Tm, Yb, Lu; *T* = Si, Ge) [8,9,10,11,12,13,14,15,16,17,18,19,20,21,22,23,24,25,26,27]. All of these exceed the scope of the 8-*N* rule and show interesting physical properties such as superconductivity. Although barium and germanium form two superconducting BaGe_3_ modifications, *hP*8 [14] and *tI*32 [15], a corresponding barium–silicon phase remained clandestine so far. In this study, we describe high-pressure high-temperature synthesis, crystal structure and chemical bonding properties of BaSi_3_ as well as the temperature dependence of electrical resistivity and magnetic susceptibility.

## 2. Materials and Methods

Barium trisilicide was prepared by high-pressure high-temperature synthesis. Sample handling except for high-pressure synthesis itself was accomplished in argon-filled glove boxes (MBraun, H_2_O and O_2_ <0.1 ppm). The precursor mixture was manufactured by arc melting of barium (Alfa Aesar, 99.9%) and silicon (Chempur, 99.999%) in the ratio 1:3 plus 1% excess of barium. The resulting material was intensely ground and placed in boron nitride crucibles before being transferred into MgO octahedra with an edge length of 14 mm. High-pressure high-temperature synthesis in a multi-anvil Walker-type module for one to five hours was realized at pressures between 12(2) and 15(2) GPa and temperatures from 800(80) and 1050(105) K before quenching to ambient temperature under load [28]. Calibrations of pressure and temperature by observing resistance changes of bismuth [29], as well as thermocouple-calibrated runs, had been conducted before the synthesis experiments.

Differential scanning calorimetry (DSC) experiments were realized in a Netzsch DSC 404 C device (Netzsch-Gerätebau GmbH, Selb, Germany) operated with heating and cooling rates of 10 K/min under argon atmosphere using corundum crucibles.

Phase designation was conducted by X-ray powder diffraction experiments with a Huber Image Plate Guinier Camera G670 (Huber Diffraktionstechnik GmbH & Co. KG, Rimsting, Germany), using Cu*K*α1 radiation, *λ* = 1.54056 Å. High-resolution X-ray diffraction experiments of BaSi_3_ were conducted with synchrotron radiation (*λ* = 0.399972(2) Å) at beamline ID22 of the European Synchrotron Radiation Facility. All crystallographic calculations including the determination of diffraction peak positions as well as lattice parameter and crystal structure solution and refinement by the Rietveld technique (Table 1 and Table 2) were performed with the WinCSD program package, version 2018 [30].

For metallographic analysis, samples were prepared by polishing with diamond powder disks (grain size 6, 3 and 0.25 μm) in paraffin. The investigation was performed with a Philips XL 30 scanning electron microscope (LaB_6_ cathode), comprising an EDAX Si(Li) detector for energy-dispersive X-ray spectroscopy (EDXS).

Electronic structure calculations and chemical bonding analysis were carried out with the experimentally determined lattice parameters and the refined atomic coordinates of an idealized crystal structure model without disorder. First, band structure calculations were performed with the TB-LMTO-ASA (TB: tight-binding, LMTO: linear muffin tin orbitals, ASA: atomic sphere approximation) program package [31]. In these computations, the Barth-Hedin exchange potential [32] was used. The following radii of the atomic spheres were applied for the calculations: *r*(Ba1) = 2.375 Å, *r*(Ba2) = 2.419 Å, *r*(Si1) = 1.430 Å, *r*(Si2) = 1.415 Å, *r*(Si3) = 1.418 Å. Due to the calculations already including corrections for the neglect of interstitial regions and partial waves of higher order [33], insertion of additional so-called empty spheres was not necessary. A basis set of Ba(6*s*,5*d*) and Si(3*s*,3*p*) orbitals was employed for self-consistent calculations with Ba(6*p*,5*d*) and Si(3*d*) functions being downfolded. To obtain the partial waves, the radial scalar-relativistic Dirac equation was solved. After convergence, the electronic density of states (DOS) was calculated using a mesh of 32 × 32 × 32 *k*-points.

For the analysis of the chemical bonding in direct space the electron density and the electron localizability indicator ELI-D was calculated [34,35] with a module implemented in the program package. The computed spatial arrangement of ELI-D and electron density was analyzed with the program DGrid [36]. For this purpose, the electron density was integrated within so-called basins, i.e., space regions confined by zero-flux surfaces of the gradient field. This technique follows the procedure proposed in the Quantum Theory of Atoms In Molecules (QTAIM [37]) and provides electron counts for the basins of atoms (QTAIM populations of the atoms) and bonds (bond populations). The combined analysis of electron density and ELI-D constitutes a basis for the description of chemical bonding [38,39], especially in intermetallic compounds [40,41].

Electrical resistivity *ρ* was measured using a cuboid (1.00 mm × 1.80 mm × 0.90 mm) cut from a polycrystalline sample of cylindrical shape by a direct-current four-probe method carried out on a PPMS (Quantum Design International, San Diego, USA) AC transport option, 0.11 to 2.0 K and 2.0 to 305 K). The inaccuracy of *ρ* was estimated to be ±20%, because of the intricate contact geometry. The measurement of magnetic susceptibility *χ* was conducted using a polycrystalline sample of cylindrical shape (diameter 1.0 mm, length 1.0 mm) and a SQUID magnetometer (MPMS XL-7, Quantum Design).

The thermal stability of the high-pressure phase was studied by differential scanning calorimetry (DSC) experiments. A commercially available Netzsch DSC 404C apparatus was equipped with corundum crucibles and operated with an argon atmosphere. Both heating and cooling were realized at a rate of 10 K/min.

## 3. Results and Discussion

**Figure 1:**
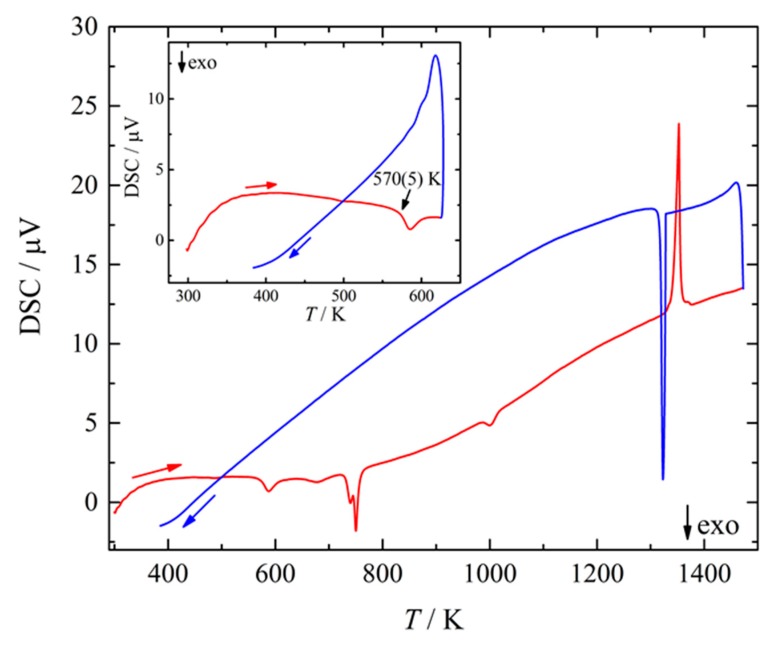
Differential scanning calorimetry (DSC) curve of BaSi_3_ taken on heating (red curve) and cooling (blue curve) in the temperature range from 300 to 1475 K with a heating rate of 10 K/min at ambient pressure under argon atmosphere. Inset: DSC curve of BaSi_3_ between 300 and 625 K illustrating the onset of the exothermal decomposition at 570(5) K.

The new phase was obtained by high-pressure high-temperature treatment of pre-reacted Ba_25_Si_75_ mixtures before quenching. The chemical composition of the hp-ht product as determined using energy dispersive X-ray spectroscopy amounted to Ba_22.4_Si_77.6_ which corresponds to BaSi_3_ within the estimated error. Differential scanning calorimetry (DSC) measurements at ambient pressure evidenced the decomposition of BaSi_3_ (Figure 1) at 570(5) K. The first exothermic anomaly upon heating (Figure 1, inset) corresponded to the onset of disintegration. Powder XRD and EDXS analyses of the obtained decomposition product after heating to 623 K evidenced the formation of (*hP*3)BaSi_2_ [42] plus two amorphous phases with averaged composition Ba_24.3(5)_Si_75.7(5)_ (≈BaSi_3_) and Ba_14.5(5)_Si_85.5(5)_ (≈BaSi_6_), respectively. The following features at 725(5), 745(10) and 985(10) K represented different reaction steps of the decomposition products of BaSi_3,_ and XRD data evidenced the formation of (*oP*24)BaSi_2_ (often labeled as Ba_2_Si_4_ [43]) and (*cF*8)Si [44]. As the final effect at 1325 K correlated with a corresponding signal upon cooling, the signal is essentially assigned to the eutectic of BaSi_2_ + Si in full accordance with phase diagram data [45]. As the cooling curve shows no further signal indicating the back transformation of BaSi_2_ and Si into BaSi_3,_ the experimental data indicate that BaSi_3_ is a high-pressure phase, which is metastable at ambient pressure.

**Figure 2:**
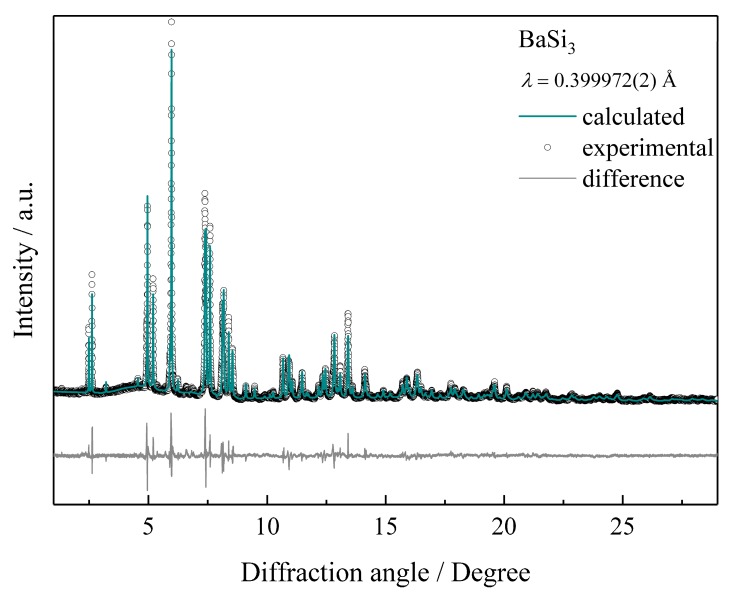
Synchrotron X-ray powder diffraction pattern of BaSi_3_ and the result of the crystal structure refinement by the Rietveld method.

Characterization of the crystal structure was performed by X-ray powder diffraction experiments using synchrotron radiation. Indexing of peak positions yields a tetragonal unit cell for the new high-pressure phase BaSi_3_. The resulting diffraction symbol, as well as the axial ratio c/a, could be compatible with a CaGe_3_-type atomic arrangement as predicted by an earlier ab-initio study [46]. However, refinements of this structure pattern in space group *I*4/*mmm* (no. 139) do not produce satisfactory results with respect to reflection intensities (which is reflected in residuals *R*(P) = 0.215). Thus, structure models in maximal non-isomorphic subgroups were developed. The refinement of a structure model in space group *I*$\bar{4}$*2m* converged in a straightforward manner (R(P) = 0.073). However, unusually large displacement parameters motivated the introduction of split-positions for Ba1 and Si3 in the final refinements (Figure 2 and Table 1 and Table 2). As the profiles of some reflections show evidence for subtle shoulders, crystal structure solutions assuming further decrease of symmetry were attempted. With the available X-ray powder diffraction data, these tests remained unsuccessful.

**Figure 3:**
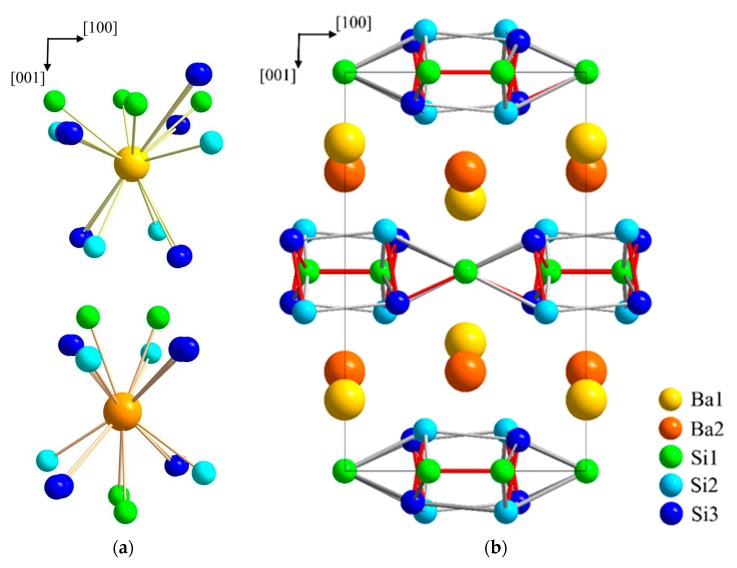
Crystal structure of BaSi_3_. (**a**) Coordination polyhedrons of Ba1 and Ba2. Both positions of the disordered atom Si3 are indicated. (**b**) Visualization of the layer-type arrangement of BaSi_3_ using average positions for the disordered atoms Ba1 and Si 3. Short silicon–silicon next-neighbor distances are indicated by red lines to emphasizes the similarity of the atomic arrangement to the CaGe_3_-type, which is adopted by a number of related trisilicides.

The crystal structure of BaSi_3_ (Figure 3) may be described as comprising silicon layers which are stacked along the *c* axis and separated by barium atoms. Ba1 and Ba2 are surrounded by 13 and 12 silicon atoms, respectively. The barium–silicon distances in the irregular polyhedrons of Ba1 and Ba2 cover the range from 3.325(8) to 3.79(1) Å and from 3.375(3) to 3.592(9) Å, respectively. For comparison, the binary silicon-rich barium compounds BaSi_6_ and BaSi_2_ exhibit Ba-Si distances in the range from 3.20(1) to 3.82 Å [47,48,49,50].

The silicon atoms occupy three distinct positions. The shortest distances *d*(Si1-Si1) of 2.34(1) Å and *d*(Si2-Si3) of 2.33(2) Å denote the occurrence of Si_2_ entities which are similar to the dumbbells occurring in CaGe_3_. However, in BaSi_3_ these primary Si2–Si3-fragments are inclined by approximately 10° with respect to the *c* axis. The tilt causes a 5° twist of the rectangular units in *b* direction with alternating rotation directions of neighboring units. The resulting distorted tetragonal prisms are linked by (Si1)_2_-dumbbells in a perpendicular orientation. As a result of the tilting, the distances *d*(Si1-Si3), which are of equal length in the CaGe_3_ type, distort into short (2.351(8) Å) and longer contacts (2.524(8) Å).

**Figure 4:**
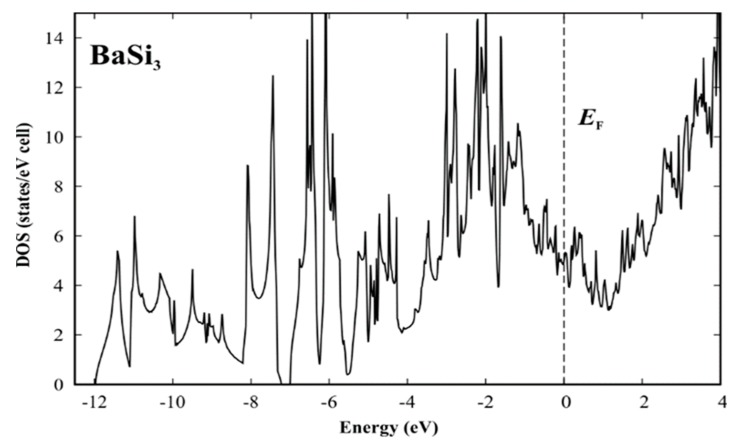
Calculated electronic density of states (DOS) for the idealized structure model [51] of BaSi_3_.

The refined crystal structure model of BaSi_3_ evidences that the new compound comprises silicon atoms in unusual connectivity situations. Assuming single-bonded silicon dumbbells would imply a rather non-realistic electron balance with huge electron demand: (Ba^2+^)_2_{[(1b)Si−(1b)Si)]^6−^}_3_ × 12p^1+^. Considering the slightly longer distances of Si1 and Si3 as additional single bonds reduces the problem: (Ba^2+^)_2_[(3b)Si1^1−^]_2_[(1b)Si2^3−^]_2_[(3b)Si3^1−^]_2_ × 6p^1+^. In agreement with this predicted electron demand, the calculated electronic density of states reveals that the Fermi level (calculated for the idealized structure model without disorder [51]) is located below the pseudogap (Figure 4). Nevertheless, a quantitative estimate of the electron count requires a more elaborate analysis of chemical bonding in real space [34,35].

By comparison with distances in analogous compounds (*d*(Si-Si) between 2.390(1) and 2.443(3) Å [8,9,10]) or modifications of elemental silicon (*d*(Si-Si) in *cI*16 from 2.3283(4) to 2.3841(4) Å [52] and in *cF*8 2.3516 Å [53]), the shortest homoatomic Si-Si contacts in BaSi_3_ (2.33(2) to 2.351(8) Å) may easily be considered as bonding. The remaining interactions in BaSi_3_ require further analysis.

**Figure 5:**
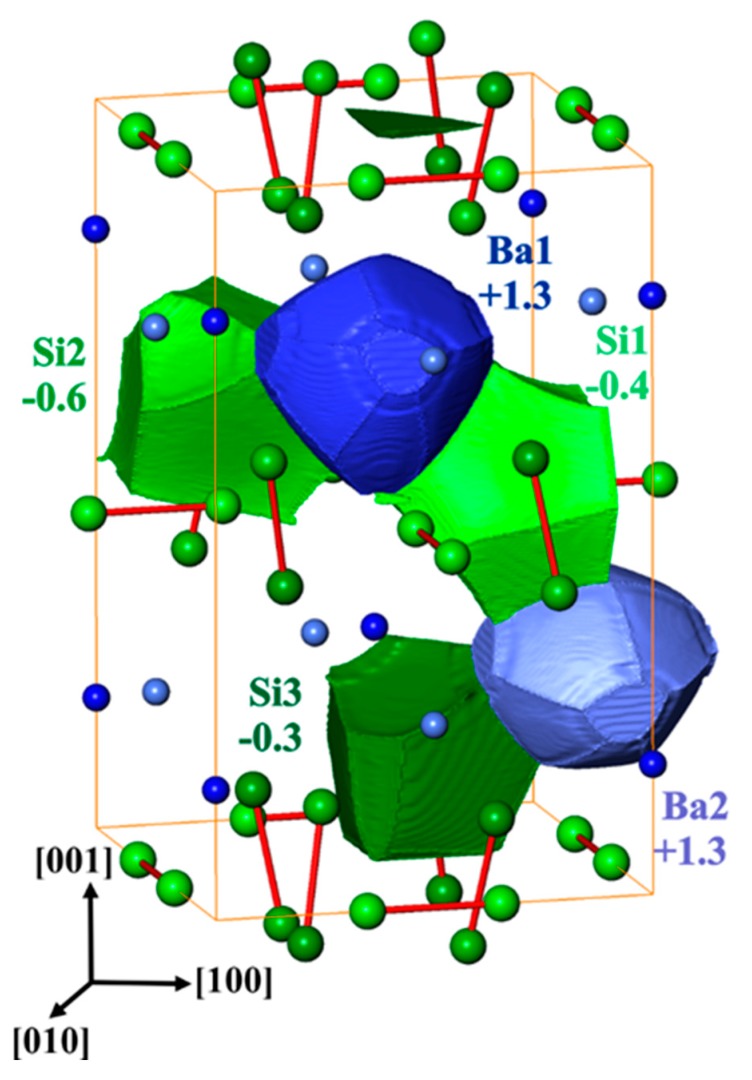
The shapes of quantum theory of atoms in molecules (QTAIM) atoms and their calculated effective charges in BaSi_3_. The numbers are in units of elementary charge.

The calculated electron density reveals Ba species with almost spherical shape indicating essentially ionic character. The shapes of the silicon species have a more polyhedral character, especially the contact faces between the two nearest silicon atoms appear rather flat, which is characteristic for non-polar covalent bonding. Integrating the electron density within the QTAIM shapes and subtracting the atomic number yields effective charges. The net charge transfer from barium to silicon (Figure 5) is in accordance with the electronegativity difference of the constituents. The effective charges of the barium species (+1.30) fall into the range of +1.2 to 1.4 which is observed for barium–germanium clathrates [54] but are markedly smaller than those of calcium in CaSi_3_ (+1.44, +1.49 [8]). Moreover, the computed charges of silicon in BaSi_3_ (−0.3 to −0.6) show a larger spread than those in CaSi_3_ (−0.40 and −0.54). Both findings consistently indicate a slightly different organization of the bonding in BaSi_3_ in comparison to the other trisilicides.

**Figure 6:**
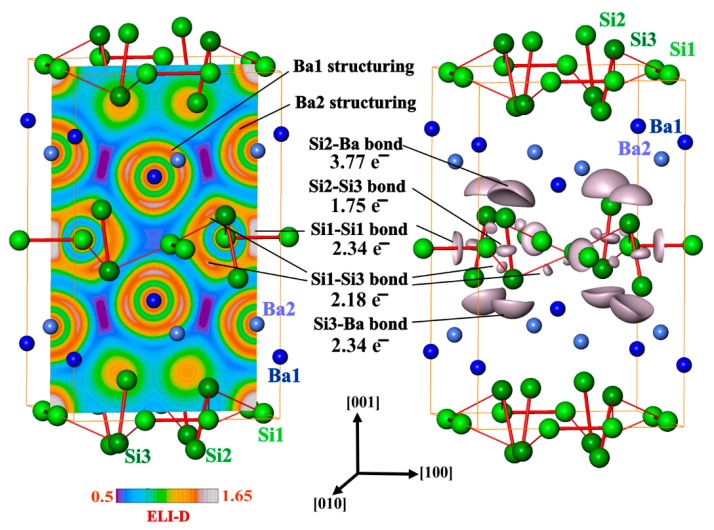
Electron localizability indicator and atomic interactions in BaSi_3_: (**left**) distribution of ELI-D in the (200) plane reveals structuring of the penultimate shells of Ba1 and Ba2 as well the Si1-Si1 bonds; (**right**) the isosurface of ELI-D for the value of 1.5 shows Si1-Si1, Si1-Si3 and Si2-Si3 bonds as well as the five-atomic bonds Si2Ba_4_ and Si3Ba_4_.

Further analysis of the chemical bonding in BaSi_3_ was realized by applying the electron localizability approach. The ELI-D distribution in the penultimate shell of the barium atoms shows significant deviations from spherical symmetry (structuring [38], Figure 6, left pannel). Quantitative characterization of this trend and comparison to the recent results for YGa_6_, YGa and *t*-Y_5_Ga_3_ [55] reveals fingerprints for the participation of the penultimate shell in the bonding interactions [38,56]. In the valence region of silicon, five different ELI-D maxima are observed. Three of them are located on (or close to) the shortest Si-Si contacts (Figure 6) indicating covalent Si-Si bonding. Two remaining ones are located on the outer side of the Si2-Si3 dumbbell suggesting lone pairs. In an isolated Si_2_ molecule, each basins of these maxima would have contacts with one core basin of silicon, i.e., it would reflect a lone pair such as in RhBi_4_ [57], CoBi_3_ [58] or *hp*-CuBi [59]). In BaSi_3_, each of these attractors represents a five-center bond SiBa_4_.

Integration of the electron density within the bonding basins (Figure 6, right pannel) yields the values 2.34 and 1.75 for the Si1-Si1 and Si2-Si3 dumbbells, respectively, as well as 2.18 for the Si1-Si3 contact. Consequently, the shortest Si-Si distances correspond 2*c*-2*e* bonds in good approximation. The calculated population of the lone-pair basin of Si3 amounts to 2.34 electrons, which is close to the value of two as predicted by the 8-*N* rule. Yet, the three-bonded Si1 atom does not show any basin resembling a lone-pair. The Si2 atom is single-bonded with a calculated population of the lone-pair basin of 3.77 electrons, which is still below the predicted six on basis of the 8-*N* rule. The analysis evidences that the interpretation of the crystal structure of BaSi_3_ as a CaGe_3_-type packing with slightly tilted Si_2_ dumbbells is insufficient.

**Figure 7:**
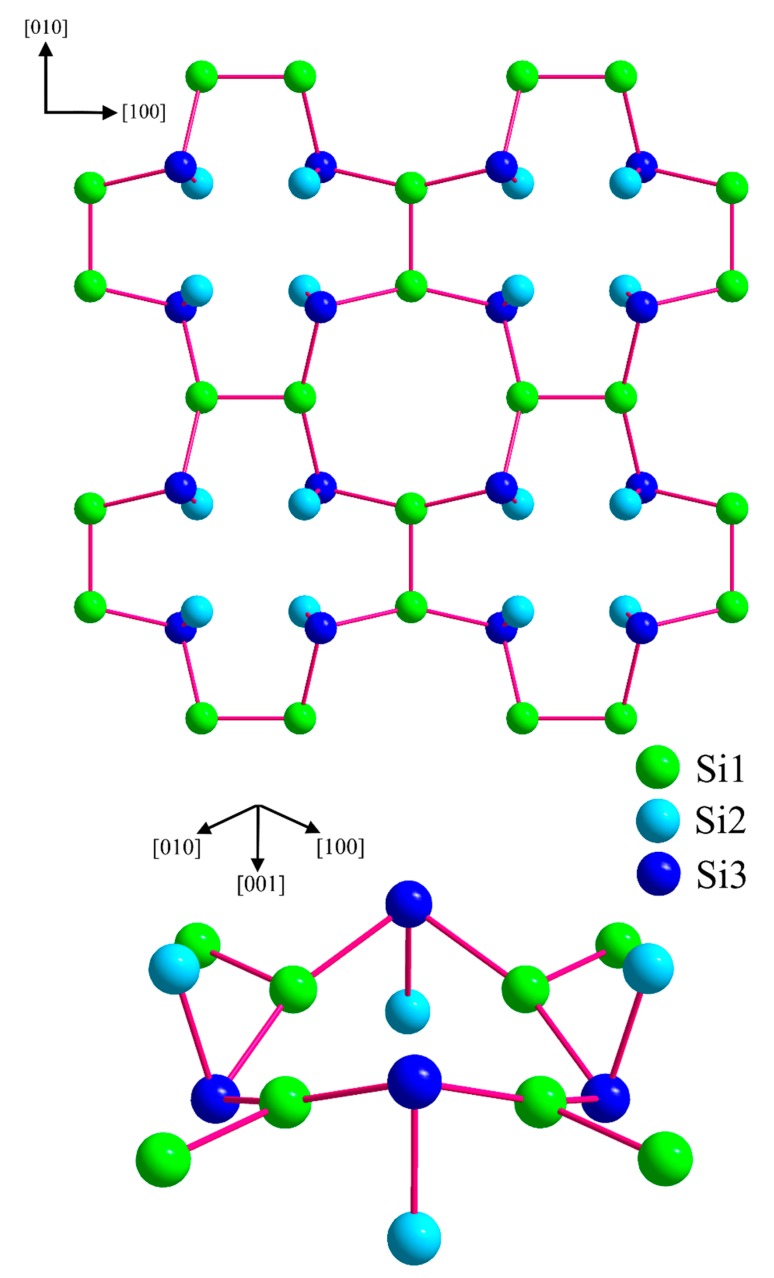
Anionic silicon partial structure in BaSi_3_. (**top**) Covalent silicon layers with the 2*c*2*e* interactions of silicon indicated by red lines, (**bottom**) detail of the partial structure for visualizing the connectivity and the orientation of the (Si1)_2_ and the (Si2-Si3) dumbbells in more detail. Please note that the disorder of Si3 is eliminated in this idealized structure model [51].

Instead, the bonding analysis yields direct evidence for a more adequate description of the atomic arrangement (Figure 7). The Si_2_ dumbbells (Si1)_2_ and Si2-Si3 are oriented in an almost perpendicular way. However, the condensation of these diatomic units proceeds exclusively via three-bonded Si1 and Si3 atoms. The Si2 atoms form a single bond to the Si3 atoms and are arranged above and below the puckered sheets formed Si1 and Si3. These special covalent segments are interconnected by barium cations interacting with the anionic substructure by polar five-center Si2Ba_4_ and Si3Ba_4_ bonds.

**Figure 8:**
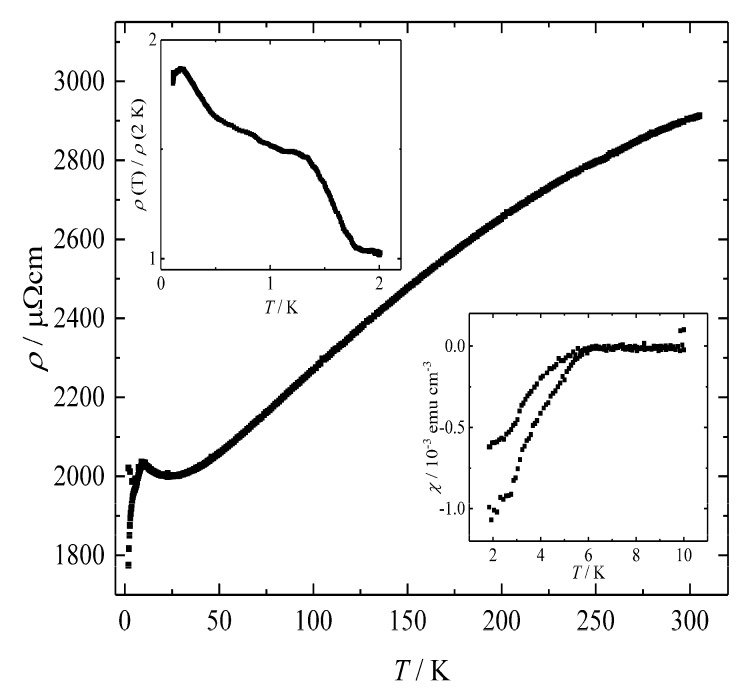
Temperature-dependent electrical resistivity *ρ* of BaSi_3_ at zero-field between 2 and 305 K. Insets: Low-temperature electrical resistivity of BaGe_3_ between 0.11 and 2.0 K at zero-field. The normalization was performed to eliminate changes originating from different measurement geometries. The second inset shows the temperature dependence of the magnetic susceptibility *χ* between 1.8 and 10 K measured in a field of 0.2 mT. The subtle decrease is attributed to a small amount of superconducting impurity, but bulk superconductivity can clearly be ruled out.

In agreement with the electron balance and the calculated band structure, electrical resistivity measurements on BaSi_3_ between 2 and 305 K show a positive slope above approximately 25 K indicating metal-type conductivity behavior (Figure 8). At room temperature and zero-field, the value for BaSi_3_ amounts to *ρ*_300 K_ = 2906 μΩ cm with a residual resistance ratio (RRR) *ρ*_293 K_/*ρ*_4 K_ = 1.5. In comparison to analogue high-pressure phases of germanium [12,13,19,27], the resistivity is high and shows only weak temperature dependence which is typical for polycrystalline silicides [60]. The changes of both resistivity and susceptibility below 6 K are attributed to traces of superconducting BaSi_2_ [61]. The little overall resistivity changes down to 110 mK in conjunction with the minor magnetic susceptibility change (Figure 8) clearly evidence that these changes do not originate from a bulk superconducting state.

## Figures and Tables

**Table 1:** Data collection, structure refinement and crystallographic information for BaSi_3_.

Composition	BaSi_3_
Space group, Pearson symbol	*I*$\bar{4}$*2m* (no. 121), *tI*32
Structure type	BaSi_3_
Unit cell parameters	
*a*/Å	7.6971(2)
*c*/Å	12.6605(3)
*V*/Å^3^	750.07(5)
Formula units per unit cell, *Z*	4
Measurement range	1.0° ≤ 2*θ* ≤ 29.0°, 0.002° step width
Measured points/reflections	14000/281
*R*(P)/*R*(wP)	0.073/0.099

**Table 2:** Wyckoff positions, site occupancy factors (SOF), relative atomic coordinates and equivalent displacement parameters *B*_iso_ (in Å) for BaSi_3_. The values of the estimated standard deviation consider the local correlations of powder diffraction data.

Atom	Site	SOF	*x*/*a*	*y*/*b*	*z*/*c*	*B*_iso_
Ba1	8*i*	0.5	0.0162(1)	*x*	0.1785(2)	0.67(2)
Ba2	4*d*	1.0	1/2	0	1/4	0.60(2)
Si1	8*f*	1.0	0.347(1)	0	0	1.37(2)
Si2	8*i*	1.0	0.332(3)	*x*	0.1012(9)	1.16(2)
Si3	16*j*	0.5	0.2078(9)	0.2282(9)	0.422(1)	1.24(2)
